# Persistency of Prediction Accuracy and Genetic Gain in Synthetic Populations Under Recurrent Genomic Selection

**DOI:** 10.1534/g3.116.036582

**Published:** 2017-01-04

**Authors:** Dominik Müller, Pascal Schopp, Albrecht E. Melchinger

**Affiliations:** Institute of Plant Breeding, Seed Sciences and Population Genetics, University of Hohenheim, 70599 Stuttgart, Germany

**Keywords:** genomic prediction, recurrent selection, synthetic populations, prediction accuracy, genetic gain, GenPred, Shared Data Resources, Genomic Selection

## Abstract

Recurrent selection (RS) has been used in plant breeding to successively improve synthetic and other multiparental populations. Synthetics are generated from a limited number of parents $\left(N_{p}\right),$ but little is known about how $N_{p}$ affects genomic selection (GS) in RS, especially the persistency of prediction accuracy ($r_{g,\hat{g}}$) and genetic gain. Synthetics were simulated by intermating $N_{p}$= 2–32 parent lines from an ancestral population with short- or long-range linkage disequilibrium ($LD_{A}$) and subjected to multiple cycles of GS. We determined $r_{g,\hat{g}}$ and genetic gain across 30 cycles for different training set (*TS*) sizes, marker densities, and generations of recombination before model training. Contributions to $r_{g,\hat{g}}$ and genetic gain from pedigree relationships, as well as from cosegregation and $LD_{A}$ between QTL and markers, were analyzed via four scenarios differing in (i) the relatedness between *TS* and selection candidates and (ii) whether selection was based on markers or pedigree records. Persistency of $r_{g,\hat{g}}$ was high for small $N_{p},$ where predominantly cosegregation contributed to $r_{g,\hat{g}}$, but also for large $N_{p},$ where $LD_{A}$ replaced cosegregation as the dominant information source. Together with increasing genetic variance, this compensation resulted in relatively constant long- and short-term genetic gain for increasing $N_{p}$ > 4, given long-range LD_A_ in the ancestral population. Although our scenarios suggest that information from pedigree relationships contributed to $r_{g,\hat{g}}$ for only very few generations in GS, we expect a longer contribution than in pedigree BLUP, because capturing Mendelian sampling by markers reduces selective pressure on pedigree relationships. Larger *TS* size ($N_{TS}$) and higher marker density improved persistency of $r_{g,\hat{g}}$ and hence genetic gain, but additional recombinations could not increase genetic gain.

RS is an integral tool in plant breeding that targets the systematic improvement of quantitative traits in broad-based populations by increasing the frequency of favorable alleles, while maintaining genetic variability (Hallauer and Carena 2012). Source materials in allogamous crops include open-pollinated and synthetic populations (synthetics, Hallauer 1992). Synthetics are created by intermating a limited number of parental components and cross-pollinating the progeny for one or several generations (Falconer and Mackay 1996). A prominent example is the Iowa Stiff Stalk Synthetic (BSSS), which was developed from 16 inbred lines in the 1930s and has since been subjected to two long-term RS programs (Hallauer 2008), which have contributed a large proportion of today’s commercial maize germplasm (Mikel and Dudley 2006).

GS is a novel statistical method (Meuwissen *et al.* 2001) with the capability to accelerate future genetic progress in plant breeding (Heffner *et al.* 2010). Several studies indicate a potential superiority of GS over phenotypic selection (Bernardo 2009; Wong and Bernardo 2009; Jannink 2010; Yabe *et al.* 2013), marker-assisted selection (Bernardo and Yu 2007; Wong and Bernardo 2009; Heffner *et al.* 2010, Yabe *et al.* 2013), as well as pedigree-based selection (Muir 2007; Wolc *et al.* 2011a, 2016; Bastiaansen *et al.* 2012; Van Grevenhof *et al.* 2012). Although the usefulness of GS across two selection cycles has empirically been demonstrated in biparental maize families (Massman *et al.* 2013; Beyene *et al.* 2015), experimental results on long-term GS are still missing.

GS has further been proposed as a particularly suitable tool for RS in synthetics (Windhausen *et al.* 2012; Gorjanc *et al.* 2016). In this context, an established prediction equation could be used repeatedly for multiple cycles of selection without retraining. Combined with the use of off-season nurseries, this promises to increase genetic gain per unit time and to reduce costs for phenotyping (Bernardo and Yu 2007). The success of this strategy largely depends on persistency of the $r_{g,\hat{g}}$ of estimated breeding values (*EBV*) across selection cycles to ensure satisfactory genetic gain when selection candidates are separated by one or more cycles from the model training generation. Although formulas for forecasting $r_{g,\hat{g}}$ in a single cycle were derived (Daetwyler *et al.* 2008; Hayes *et al.* 2009; Goddard 2009; Goddard *et al.* 2011), no closed analytical solutions are available for calculating $r_{g,\hat{g}},$ the additive genetic variance ($\sigma_{A}^{2}$) and the cumulative genetic gain (∑ΔG) across several selection cycles. This is because changes in the LD pattern, allele frequencies, and loss of polymorphisms are unpredictable (Jannink 2010).

While empirical results on persistency of $r_{g,\hat{g}}$ in actual plant breeding programs are scarce to date, several simulation studies across multiple generations investigated $r_{g,\hat{g}}$ of GS, assuming random mating of the whole population between generations (Meuwissen *et al.* 2001; Habier *et al.* 2007; Nielsen *et al.* 2009; Solberg *et al.* 2009). Others assumed selection and were therefore able to evaluate potential genetic gain using GS (Muir 2007; Sonesson and Meuwissen 2009; Jannink 2010; Bastiaansen *et al.* 2012; Yabe *et al.* 2013, 2016; Liu *et al.* 2015). However, these studies generally considered fairly large effective population sizes $N_{e}\geq 100,$ which are unrealistic for synthetics in plant breeding. In synthetics, the number of parents is usually relatively small and parents are often related, leading to small $N_{e}$ of the population. It is yet unclear how such a small $N_{e}$ influences the persistency of $r_{g,\hat{g}}$ in genomic RS.

Initially, LD between QTL and molecular markers (commonly SNPs) of high density maps was considered as the only source of information exploited in GS (Meuwissen *et al.* 2001). In synthetics, LD between QTL and SNPs is attributable to (i) $LD_{A}$ in the population from which the parents were taken, and (ii) sample LD, randomly generated by using a restricted number of parents $N_{p}$ (Schopp *et al.* 2017). Sample LD is conserved from parents to progeny between cosegregating loci, and has therefore been termed cosegregation. However, it was also demonstrated that SNPs contribute to $r_{g,\hat{g}}$ by capturing pedigree relationships between individuals (Habier *et al.* 2007). Research in a companion paper (Schopp *et al.* 2017) showed that the choice of $N_{p}$ in synthetics crucially affects the relative importance of $LD_{A}$ and cosegregation as well as the contribution of pedigree relationships in a single cycle of GS in synthetics. However, no study systematically investigated the importance of these information sources for the persistency of $r_{g,\hat{g}}$ and $\sum^{\text{​}}\text{Δ}G$ in recurrent GS.

Besides the choice of $N_{p},$ an important question is how often the source material should be recombined before starting RS. Additional recombination might release genetic variability useful for long-term genetic gain (Schnable *et al.* 1996). For instance, Bernardo (2009) recommended the use of F_2_ instead of F_1_ plants in the production of maize doubled haploids. However, additional recombination might also adversely affect the three information sources in GS, and so far studies have not addressed whether this can outweigh the potential increase in long-term genetic gain.

In the present study, we applied fully stochastic forward-in-time simulations and generated two ancestral populations differing substantially in $LD_{A}.$ From these, we sampled different numbers of parents $N_{p}$ to create synthetics that were subjected to multiple cycles of recurrent GS, either directly or after additional generations of recombination. Our objectives were to (i) analyze $r_{g,\hat{g}}$ and $\sum^{\text{​}}\text{Δ}G$ in recurrent GS, depending on the number of parents $N_{p},$ $LD_{A},$ and the number of recombination generations $N_{R},$ and (ii) determine the importance of the three information sources, considering also $N_{TS}$ and SNP density. Finally, we discuss implications for practical decisions in breeding programs employing recurrent GS.

## Methods

### Genome properties and simulation of ancestral populations

Properties of the genome, construction of the genetic map, and simulation of ancestral populations are detailed in Schopp *et al.* (2017). In brief, we selected maize (*Zea mays L*.) as a model species using genetic map positions for 37,286 SNPs distributed over 10 chromosomes with 1913 cM in total. Using the software *QMSim* (Sargolzaei and Schenkel 2009), we simulated two ancestral populations with either short-range LD_A_ (*SR*) or extensive long-range LD_A_ (*LR*). First, we generated an initial population of 1500 diploid individuals by sampling alleles at each (biallelic) locus independently from a Bernoulli distribution with probability 0.5. Second, 5000 loci were randomly sampled from all SNPs and henceforth interpreted as QTL; all remaining loci were considered as SNP markers. Third, these individuals were randomly mated for 3000 generations with a constant population size of 1500 and a mutation rate of $2.5*10^{-5}$ until mutation-drift-equilibrium was reached. Fourth, a strong population bottleneck was imposed by reducing the population size to 30 arbitrarily selected individuals, followed by 15 additional generations of random mating to generate extensive long-range LD_A_. Lastly, the population was expanded to 10,000 individuals and randomly mated three times more to establish ancestral population *LR*. Ancestral population *SR* was derived from *LR* by continuing random mating for 100 generations with constant population size of 10,000 to break down long-range LD_A_. Due to this large population size, genetic drift had only a negligible influence and hence allele frequencies were nearly identical in both ancestral populations. The heterozygous ancestral populations (*LR* and *SR*) were considered as unrelated and were used as reference bases for the pedigree of all subsequently derived individuals.

### Simulation of synthetic populations

**Figure 1:**
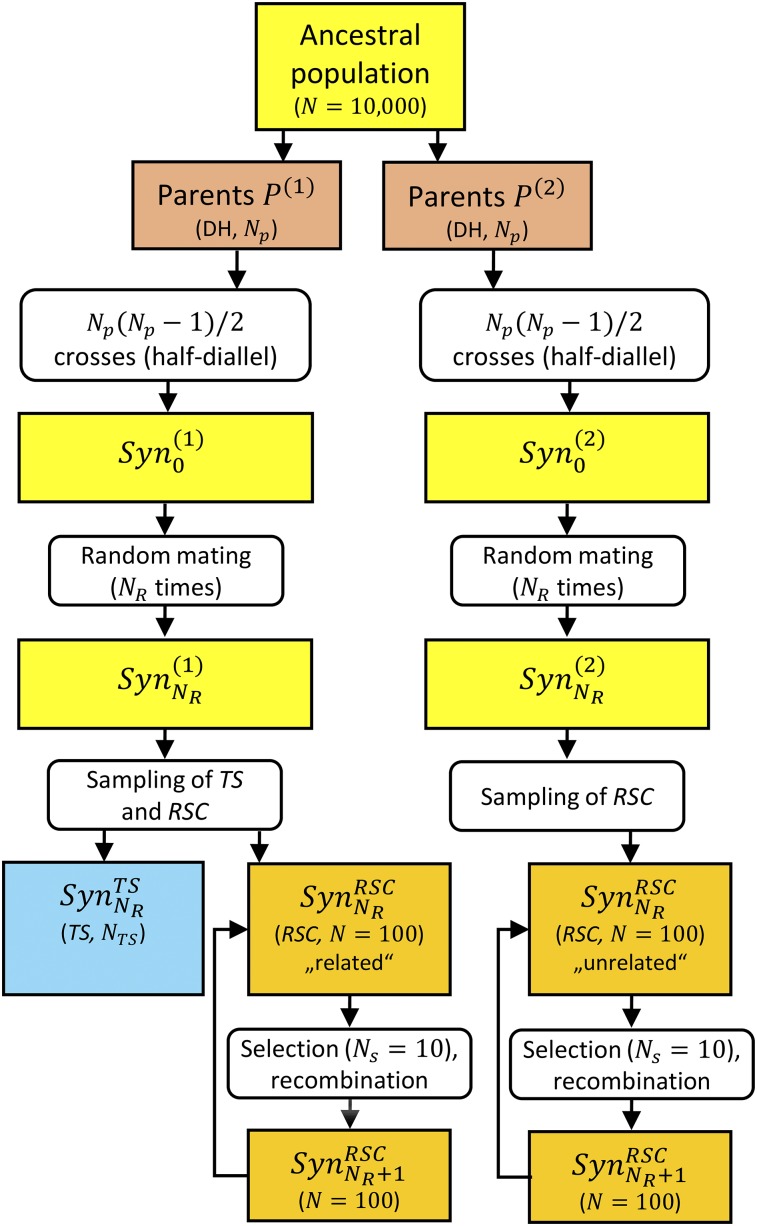
Schematic representation of the breeding program applied in this study. Two synthetic populations $Syn_{N_{R}}^{\left(1\right)}$ and $Syn_{N_{R}}^{\left(2\right)}$ were separately created by using $N_{R}$ recombination generations from $N_{p}$ parental gametes drawn from one ancestral population [with short- (*SR*) or long-range linkage disequilibrium (*LR*)]. If the training set (*TS*) and the recurrent selection candidates (*RSC*) were related, *TS* and *RSC* were sampled from the same synthetic $Syn_{N_{R}}^{\left(1\right)},$ and if they were unrelated, they were drawn from separate synthetics $Syn_{N_{R}}^{\left(1\right)}$ and $Syn_{N_{R}}^{\left(2\right)}.$ In each cycle of recurrent selection, $N_{s}=10$ individuals were selected and recombined to establish the next generation.

The RS breeding scheme applied is shown in Figure 1 and factors analyzed are listed in Table 1. The simulation of the synthetics varied, depending on whether the parents of the *TS* and the recurrent selection candidates (*RSC*) were identical ($P_{TS}=P_{RSC}$) or disjoint $\left(P_{TS}\cap P_{RSC}=ø\right).$ For $P_{TS}=P_{RSC},$ a single synthetic was simulated from which both the *TS* and the *RSC* were sampled, whereas for $P_{TS}\cap P_{RSC}=ø$ *TS* and *RSC* were taken from two synthetics having no parents in common. In both cases, $N_{p}\in \left\{2,3,4,6,8,12,16,32\right\}$ parental gametes were randomly drawn from the same ancestral population and chromosomes were doubled *in silico* to generate fully homozygous parent lines. These were intermated to obtain all possible $\left[N_{p}\left(N_{p}-1\right)\right]/2$ single crosses, denoted as generation $Syn_{0}.$ Subsequently, single crosses were randomly mated $N_{R}$ times (allowing for selfings) to obtain generation $Syn_{N_{R}},$ from which the *TS* ($Syn_{N_{R}}^{TS}$) and *RSC* ($Syn_{N_{R}}^{RSC}$) were later drawn. Here, $N_{R}\in \left\{1,2,3,4,5\right\}$ counts the number of recombination generations conducted prior to initiating RS. For the special case of $N_{p}=2,$ the $Syn_{0}$ corresponded to a F_1_ cross and $Syn_{1}$ to a F_2_ family.

**Table 1:** Overview of the factors analyzed in our simulation study

Factors	Levels
Primary factors	
Ancestral population	*SR*, *LR*
Information scenario	$Re–LD_{A}–SNP,$$Re–LD_{A}–Ped,$$Re–LE_{A}–SNP,$$Un–LD_{A}–SNP$	
Number of parents ($N_{P}$)	2, 3, 4, 6, 8, 12, 16, 32
Secondary factors	
Selection scenario	***EBV***, *TBV*, *RBV*
Number of recombination generations ($N_{R}$)	**1**, 2, 3, 4, 5
Marker density	0.125, **2.5** ${\text{cM}}^{-1}$
Training set size ($N_{TS}$)	**250**, 1000

For secondary factors, bold face type factor levels indicate the default simulation setting. *SR*, short-range; *LR*, long-range; *Re*, related; *LD_A_*, ancestral linkage disequilibrium; SNP, single nucleotide polymorphism; *Ped*, pedigree; *LE_A_*, ancestral linkage equilibrium; *Un*, unrelated; *EBV*, estimated breeding values; *TBV*, true breeding values; *RBV*, random breeding values.

### Genetic model

We assumed a quantitative trait based on 1000 biallelic QTL with purely additive gene action and absence of QTL × year interactions. For each simulation replicate, QTL were randomly sampled from the 37,286 SNPs present in the ancestral population. Following Meuwissen *et al.* (2001), absolute values of QTL effects were drawn from a gamma distribution with scale and shape parameter of 0.4 and 1.66, respectively. Signs of QTL effects were sampled from a Bernoulli distribution with probability 0.5. Although we assumed biallelic QTL, the alleles of neighboring QTL are strongly correlated due to $LD_{A}$ and linkage, effectively leading to haploblocks that could be considered as higher-level multi-allelic QTL. The true breeding value (*TBV*)$\;g_{i}$ for any individual i (either from the synthetics or from the ancestral populations) was computed as $g_{i}=\;\sum_{k=1}^{m}W_{ij\;}a_{j},$ where $W_{ij\;}$ counts the number of minor alleles at the j-th QTL centered by the respective ancestral allele frequency in *LR*, and $a_{j}$ is the associated QTL effect. Phenotypes $y_{i}$ were simulated as $y_{i}=g_{i}+e_{i},$ where $e_{i}∼N\left(0,\sigma_{e}^{2}\;\right)$ is an environmental noise variable. The error variance $\sigma_{e}^{2}$ was assumed to be constant throughout all simulations and was determined as follows: for all individuals in the ancestral population *LR*, *TBV*s were calculated according to the above procedure under replicated sampling of 1000 QTL together with their associated effects. The variance of the noise variable $\sigma_{e}^{2}$ was then set equal to the mean additive genetic variance $\sigma_{A}^{2}\left(anc\right)$. As the allele frequencies in both ancestral populations were virtually identical, $\sigma_{A}^{2}\left(anc\right)$ was also the mean additive genetic variance in ancestral population *SR*. This approach implies that the heritability in ancestral populations *LR* and *SR* was, on average, 0.5. Heritability was lower in the synthetics due to the finite sample of parents and, on average, $h^{2}\to 0.5$ for $N_{p}\to 20,000.$

### Information source scenarios

We employed four distinct scenarios to evaluate the contributions of the three information sources used in Genomic Best Linear Unbiased Prediction (GBLUP) for estimating actual relationships at causal loci by SNPs (*cf*. Habier *et al.* 2013). These scenarios can be distinguished by (i) the relatedness of the *TS* and *RSC* and (ii) the type of data employed for calculating the relationship matrix used as a kernel in GBLUP (Supplemental Material, Table S1).

Our standard scenario was $Re–LD_{A}–SNP,$ where the *TS* and *RSC* were related (Re) as their parents were identical $\left(P_{TS}=P_{RSC}\right).$ The kernel in GBLUP was calculated based on SNPs (excluding QTL) and thus contained genomic relationships. As a consequence, this scenario harnesses all three sources of information, namely: (i) pedigree relationships captured by SNPs, (ii) cosegregation between QTL and SNPs by virtue of the parents being identical, and (iii) $LD_{A}$ between QTL and SNPs due to the presence of $LD_{A}$ in the ancestral population, which was carried over to the synthetics. *$Re–LD_{A}–SNP$* is a realistic scenario and is perhaps the most frequent scenario encountered in applications of GS.

Scenario $Re–LE_{A}–SNP$ was artificial and was derived from $Re–LD_{A}–SNP.$ Here, for each of the 10 chromosomes, the multi-locus genotypes of QTL and SNPs were regarded as separate units and were reshuffled among the $N_{p}$ parents prior to intermating. This procedure broke up historical associations between QTL and SNPs due to $LD_{A},$ while conserving the LD structure among QTL and among SNPs as well as their allele frequencies. Hence, information from $LD_{A}$ cannot contribute to $r_{g,\hat{g}}$ and any LD between QTL and SNPs is exclusively due to sampling a limited number of parental gametes from the ancestral population, *i.e.*, sample LD.

Scenario $Re–LD_{A}–Ped$ was identical to $Re–LD_{A}–SNP$ except that the kernel of GBLUP was the numerator relationship matrix calculated from pedigree records of all individuals (pedigree BLUP). This scenario provided a reference for $r_{g,\hat{g}}$ and its dynamics across cycles that can be obtained exclusively from known pedigree relationships between *TS* and *RSC*.

In scenario $Un–LD_{A}–SNP$, the *TS* and *RSC* were unrelated (Un), because their parents were distinct $\left(P_{TS}\cap P_{RSC}=ø\right).$ Thus, the influence of pedigree relationships captured by SNPs and cosegregation between QTL and SNPs is eliminated, and the only remaining connection between the *TS* and *RSC* is the LD shared due to their common ancestral population, *i.e.*, $LD_{A}.$

### Genomic prediction model

We used GBLUP to predict breeding values $g_{i}$ according to the model equation

$$y_{i}=\mu +g_{i}+\epsilon_{i},$$

where $y_{i}$ and $g_{i}$ are the phenotypic and breeding values, respectively, of the i-th individual, μ is the overall population mean, and $\epsilon_{i}$ the associated model residual. Standard assumptions about the distribution of the random effects were $\left(g_{i}\right)∼MVN\left(0,\sigma_{a}^{2}K\right),$ $\left(\epsilon_{i}\right)∼MVN\left(0,\sigma_{\epsilon }^{2}I\right)$, and stochastic independence of $\left(g_{i}\right)$ and $\left(\epsilon_{i}\right).$ Variance component estimates for $\sigma_{a}^{2}$ and $\sigma_{\epsilon }^{2}$, as well as predicted breeding values were calculated using the *R*-package *rrBLUP* (Endelman 2011). The matrix $\sigma_{a}^{2}K=\left(\sigma_{a}^{2}k_{ij}\right)$ describes the variance–covariance structure of the breeding values of all individuals (TS and RSC) and was computed based on different types of data, depending on the information scenario. For $Re–LD_{A}–SNP,$ $Re–LE_{A}–SNP$, and $Un–LD_{A}–SNP,$ SNP-based genomic relationship coefficients $k_{ij}$ between individuals i and j were computed following VanRaden (2008) as

$$k_{ij}=\frac{\sum_{k}\left(x_{ik}-2p_{k}\right)\left(x_{jk}-2p_{k}\right)}{\sum_{k}2p_{i}\left(1-p_{k}\right)},$$

where $x_{ik},x_{jk}\in \left\{0,1,2\right\}$ are the genotypic SNP scores and $p_{k}$ is the frequency at the k-th SNP marker in the ancestral populations. In scenario $Re–LD_{A}–Ped,$ pedigree relationships were computed from the complete pedigree records of all individuals using the *R*-package *pedigree* (Coster 2013).

### Recurrent genomic selection scheme

The *TS* was sampled once from synthetic $Syn_{N_{R}}^{\left(1\right)}$ (Figure 1) and thereupon was used to predict breeding values in all of 30 selection cycles. The initial 100 RS candidates were sampled from the remaining individuals of $Syn_{N_{R}}^{\left(1\right)},$ if $P_{TS}=P_{RSC},$ or from the second synthetic $Syn_{N_{R}}^{\left(2\right)},$ if $P_{TS}\cap P_{RSC}=ø.$ In each cycle C, the top $N_{s}=10$ individuals were selected (before flowering) either based on (i) *EBV* calculated by GBLUP or pedigree BLUP (scenario $Re–LD_{A}–Ped$), (ii) *TBV*, corresponding to phenotypic selection with $h^{2}=1,$ or (iii) “random breeding values” (*RBV*), being chosen at random. While *EBV* shows the realistic decay of $r_{g,\hat{g}}$ (taking into account that $r_{g,\hat{g}}$ in earlier cycles influences $r_{g,\hat{g}}$ in later cycles), *TBV* provides an identical and constant selection accuracy of one, independent of $r_{g,\hat{g}}$ for all scenarios. *RBV* shows the decay of $r_{g,\hat{g}}$ without directional selection, *i.e.*, the decay that is caused by recombination and genetic drift alone. The selected fraction of 10% is realistic for practical applications and has been used in other simulation studies (*e.g.*, Jannink 2010). The selected candidates were subsequently recombined by random mating to create 100 new progeny, serving as RSC in the next selection cycle. The effects of $N_{TS}\in \left\{250,1000\right\}$ and of SNP density {0.125, 2.5 SNPs per cM} were examined in independent simulations, with default values of $N_{TS}=250$ and $2.5\;{\text{ cM}}^{-1}$ SNPs. For each combination of factors, we conducted 500 independent simulation replicates. Here, one replicate encompasses: (i) sampling of $N_{p}$ parents from the ancestral population; (ii) sampling of 1000 QTL together with their QTL effects and an appropriate number of SNPs to reach the desired marker density; (iii) creation of the synthetics assuming different numbers of generations of random mating, and sampling of the *TS* and the initial *RSC*; (iv) simulation of phenotypes for *TS* individuals; and (v) conduction of recurrent GS without retraining for 30 selection cycles. All simulations were performed with the *R* statistical language (R Core Team 2015) and code is provided in File S2.

### Cumulative genetic gain, additive genetic variance, and prediction accuracy

In each selection cycle, the cumulative genetic gain (∑ΔG) was computed as the average of all 100 *TBV*s $g_{i}$ of the *RSC* relative to the average in C=0. The $\sigma_{A}^{2}$ of the *RSC* was computed as the variance of $g_{i}$ values. The ∑ΔG was expressed in units of $\sigma_{A}(anc)$ and $\sigma_{A}^{2}$ in units of $\sigma_{A}^{2}\left(anc\right).$ $r_{g,\hat{g}}$ was calculated as the Pearson correlation coefficient between *TBV*s $g_{i}$ and predicted breeding values ${\hat{g}}_{i}$ of the *RSC*.

### Data availability

The authors state that all data necessary for confirming the conclusions presented in the article are represented fully within the article.

## Results

### Dynamics of genetic gain, prediction accuracy, and additive genetic variance

**Figure 2:**
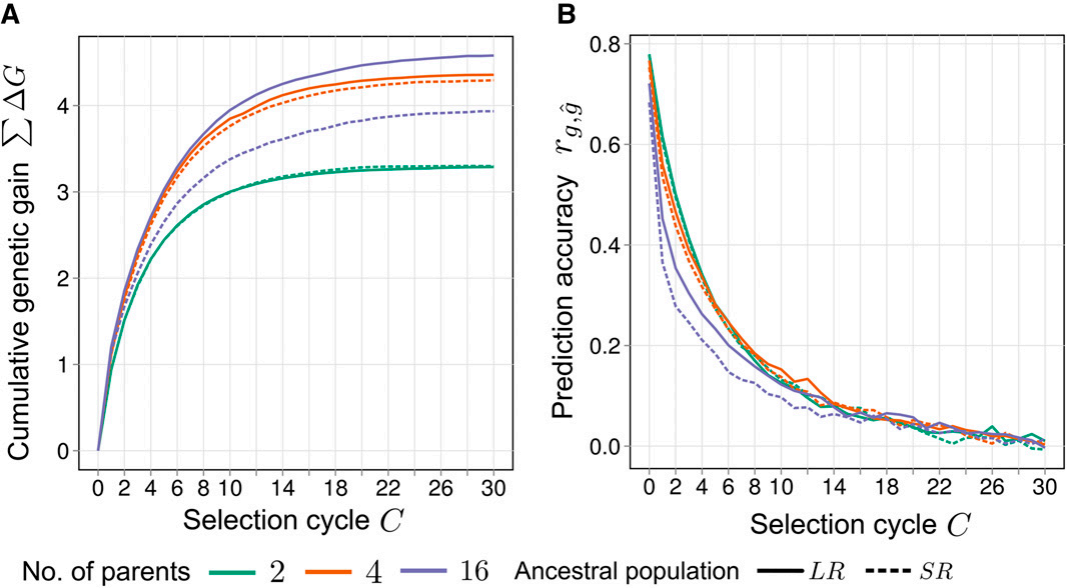
(A) Average cumulative genetic gain ∑ΔG and (B) average prediction accuracy $r_{g,\hat{g}}$ in scenario $Re–LD_{A}–SNP$ under recurrent genomic selection across C=0,1,…,30 selection cycles for synthetics produced from $N_{p}=2,\;4,\;16$ parents taken from ancestral populations *SR* or *LR*. Values of ∑ΔG are expressed in units of $\sigma_{A}\left(anc\right).$ *LD_A_*, ancestral linkage disequilibrium; *LR*, long-range linkage disequilibrium; *Re*, related; SNP, single nucleotide polymorphism; *SR*, short-range linkage disequilibrium.

An overview of the dynamics of cumulative genetic gain ∑ΔG and prediction accuracy $r_{g,\hat{g}}$ under recurrent GS for the standard scenario $Re–LD_{A}–SNP$ is given in Figure 2. Across selection cycles, ∑ΔG increased concavely, approaching a plateau. Regardless of the number of parents $N_{p},$ ∑ΔG was higher in *LR* compared to *SR*. For *LR*, ∑ΔG increased together with $N_{p},$ whereas for *SR*, ∑ΔG was lowest for $N_{p}=2,$ highest for $N_{p}=4$, and intermediate for $N_{p}=16.$ In the model training generation (C=0), $r_{g,\hat{g}}$ ranged between 0.7 and 0.8 and was higher for smaller $N_{p}.$ After the first round of selection, there was a substantial decline in $r_{g,\hat{g}}$ that was strongest for large $N_{p}.$ $r_{g,\hat{g}}$ generally approached an asymptotic value of ∼0.1 in cycle C=30. The overall level of $\sigma_{A}^{2}$ (Figure S1) in the *RSC* was higher for larger $N_{p}$ and strongly declined during selection, especially after the first cycle. In C=0, $\sigma_{A}^{2}$ was nearly identical for *LR* and *SR*, and showed a slightly steeper decline in *LR*.

### Cumulative genetic gain

**Figure 3:**
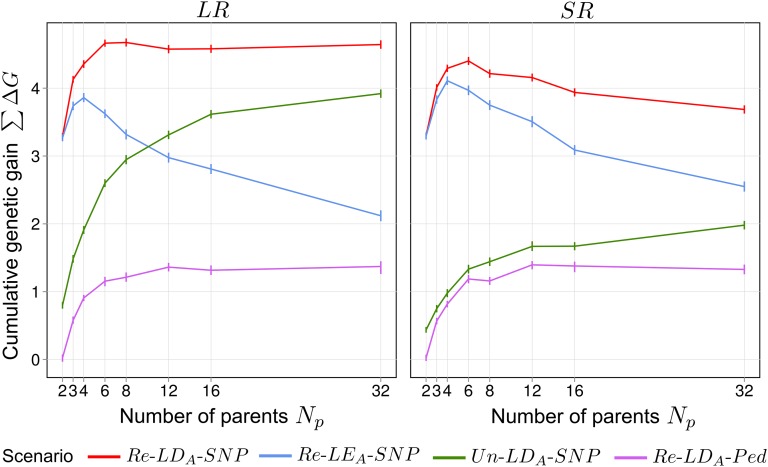
Average cumulative genetic gain ∑ΔG under recurrent genomic selection in selection cycle C=30 for synthetics produced from different numbers of parents $N_{p}$ taken from ancestral populations *SR* or *LR*. All values are expressed in units of $\sigma_{A}\left(anc\right).$ $\sigma_{A}^{2}\left(anc\right)$, mean additive genetic variance; *LD_A_*, ancestral linkage disequilibrium; *LE_A_*, ; *LR*, long-range linkage disequilibrium; *Ped*, pedigree; *Re*, related; SNP, single nucleotide polymorphism; *SR*, short-range linkage disequilibrium.

To explore in greater detail ∑ΔG in C=30 and the information sources primarily exploited, we varied $N_{p}$ between 2 and 32 (Figure 3). Here, the relationship between ∑ΔG and $N_{p}$ in scenario $Re–LD_{A}–SNP$ was strongly affected by the level of $LD_{A}.$ For *LR*, ∑ΔG initially increased between $N_{p}=2$ and $N_{p}=8$ and then remained nearly constant for larger $N_{p}.$ For *SR*, ∑ΔG also increased initially, but then strongly decreased for larger $N_{p}.$ In scenario $Un–LD_{A}–SNP$ $\left(P_{TS}\cap P_{RSC}=ø\right),$ ∑ΔG was much lower than in $Re–LD_{A}–SNP$ and monotonically increased with growing $N_{p}.$ This increase and the overall level of ∑ΔG was much higher in *LR* than *SR*. In scenario $Re–LD_{A}–Ped,$ ∑ΔG was zero for $N_{p}=2,$ and strongly increased with $N_{p},$ plateauing at $8\leq N_{p}\leq 12.$ For scenario $Re–LD_{A}–Ped,$ virtually no further genetic gain could be realized after C=2 (Figure S2).

### Persistency of prediction accuracy

**Figure 4:**
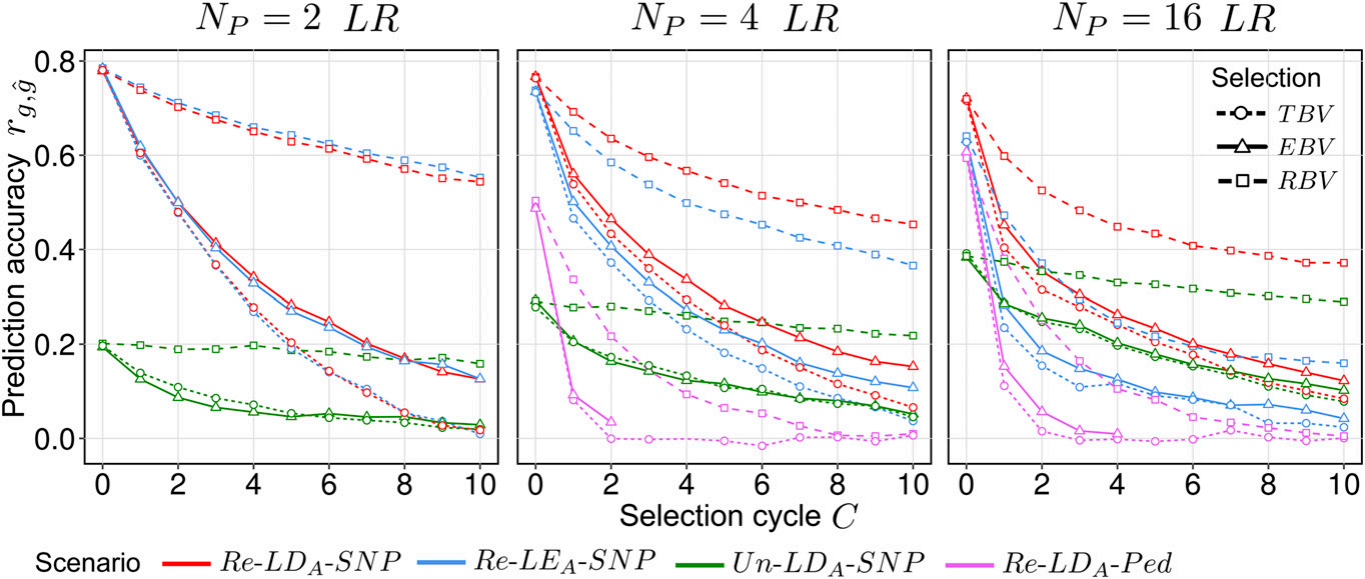
Average prediction accuracy $r_{g,\hat{g}}$ under recurrent genomic selection across C=0,1,…,10 selection cycles for synthetics produced from $N_{p}=2,4,16$ parents taken from ancestral population *LR*. Selection of candidates was based on either true breeding values (*TBV*), random breeding values (*RBV*), or estimated breeding values (*EBV*). *LD_A_*, ancestral linkage disequilibrium; *LE_A_*, ; *LR*, long-range linkage disequilibrium; *Ped*, pedigree; *Re*, related; SNP, single nucleotide polymorphism.

The persistency of $r_{g,\hat{g}}$ for selection regimes *EBV*, *TBV*, and *RBV* under *LR* is shown in Figure 4. For scenarios $Re–LD_{A}–SNP$ and $Re–LE_{A}–SNP,$ the overall level of $r_{g,\hat{g}}$ declined with growing $N_{p},$ whereas it increased for scenario $Un–LD_{A}–SNP$ (compare Figure S3). In scenario $Re–LD_{A}–SNP,$ the decay of $r_{g,\hat{g}}$ was strongest in the first selection cycle, especially for large values of $N_{p}.$ In scenario $Re–LD_{A}–Ped,$ $r_{g,\hat{g}}$ could not be calculated for $N_{p}=2$ and $N_{R}=1,$ as discussed in File S1; for $N_{p}>2,$ $r_{g,\hat{g}}$ started in C=0 at intermediate values of ∼0.5 for $N_{p}=4$ and ∼0.6 for $N_{p}=16$ but declined to zero within a few cycles if the selection was based on either *EBV* or *TBV*. With selection based on *RBV*, $r_{g,\hat{g}}$ approached zero only for C >10. Scenarios $Re–LD_{A}–SNP$ and $Re–LE_{A}–SNP$ showed identical $r_{g,\hat{g}}$ for $N_{P}=2.$ For $N_{p}>2$, $r_{g,\hat{g}}$ decreased faster in $Re–LE_{A}–SNP$ than in $Re–LD_{A}–SNP$ and more so with increasing $N_{p}.$ When ancestral long-range LD_A_ was absent (*SR*), the differences between $Re–LE_{A}–SNP$ and $Re–LD_{A}–SNP$ were generally much smaller, but otherwise trends were similar (results not shown). Scenario $Un–LD_{A}–SNP$ showed an overall low level of $r_{g,\hat{g}},$ especially for *SR*, where it was close to zero. However, the decline of $r_{g,\hat{g}}$ across cycles was attenuated compared to the other scenarios. When selection was exercised based on *TBV*, the decay of $r_{g,\hat{g}}$ was similar to selection based on *EBV*, but much stronger compared with selection based on *RBV*.

### TS size and SNP density

**Figure 5:**
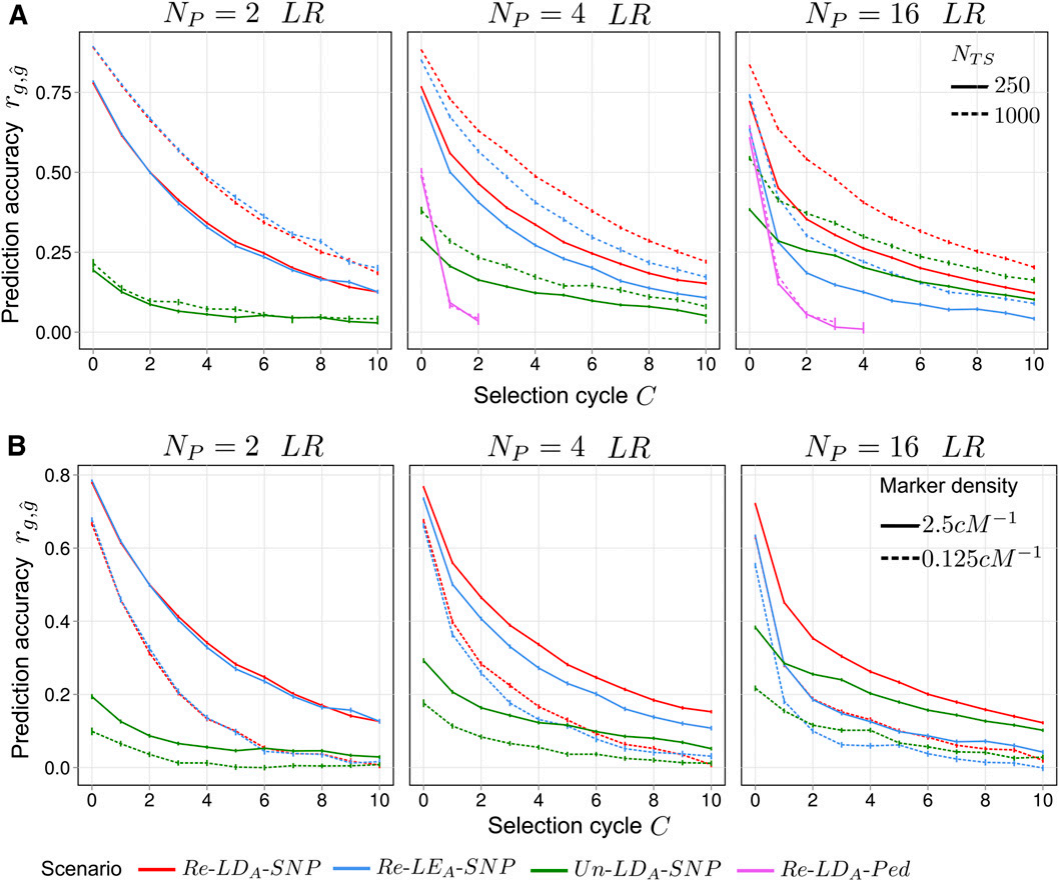
Average prediction accuracy $r_{g,\hat{g}}$ under recurrent genomic selection across C=0,1,…,10 selection cycles depending on (A) training set size $N_{TS}$ and (B) marker density for synthetics produced from $N_{p}=2,4,16$ parents taken from ancestral population *LR*. *LD_A_*, ancestral linkage disequilibrium; *LE_A_*, ; *LR*, long-range linkage disequilibrium; *Ped*, pedigree; *Re*, related; SNP, single nucleotide polymorphism.

The influence of $N_{TS}$ and SNP density on $r_{g,\hat{g}}$ under selection based on *EBV* is shown in Figure 5. For all scenarios, increasing $N_{TS}$ elevated the level of $r_{g,\hat{g}}$ across cycles. Specifically, for scenarios assuming $P_{TS}=P_{RSC},$ increasing $N_{TS}$ reduced the drop in $r_{g,\hat{g}}$ after the first selection cycle, which was not observed for scenario $Un–LD_{A}–SNP$ $\left(P_{TS}\cap P_{RSC}=ø\right).$ Increasing marker density from 0.125 to 2.5 ${\text{cM}}^{-1}$ notably increased the level of $r_{g,\hat{g}}$ for all SNP-based scenarios and led to higher persistency of $r_{g,\hat{g}}$ for SNP-based scenarios with identical parents $\left(P_{TS}=P_{RSC}\right).$ Scenario $Un–LD_{A}–SNP$ did not show an increased persistency with higher marker density.

### Number of recombinations

**Figure 6:**
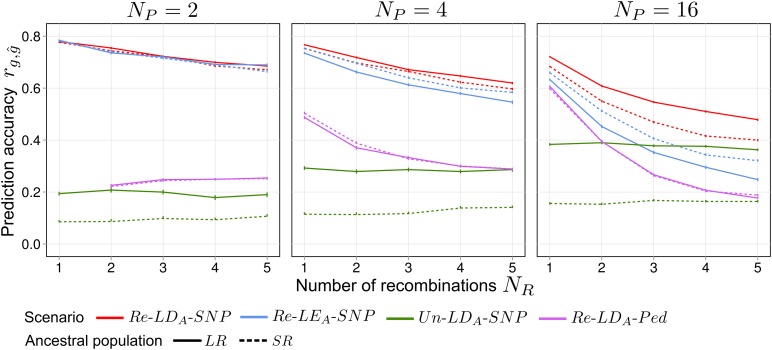
Average prediction accuracy $r_{g,\hat{g}}$ in selection cycle C=0 for different numbers of recombination generations $N_{R}$ used for production of synthetics from $N_{p}=2,4,16$ parents taken from ancestral populations *SR* or *LR*. *LD_A_*, ancestral linkage disequilibrium; *LE_A_*, ; *LR*, long-range linkage disequilibrium; *Ped*, pedigree; *Re*, related; SNP, single nucleotide polymorphism; *SR*, short-range linkage disequilibrium.

**Figure 7:**
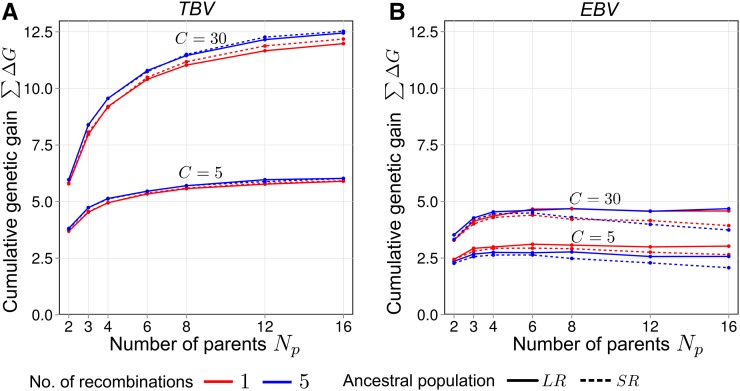
Average cumulative genetic gain ∑ΔG under recurrent genomic selection in selection cycle C=5 and C=30 for synthetics produced from different numbers of parents $N_{p}$ taken from ancestral populations *SR* or *LR* for $N_{R}=1$ and $N_{R}=5$ recombination generations. (A) Selection based on true breeding values (*TBV*), averages across all information scenarios (because values are expected to be identical). (B) Selection based on estimated breeding values (*EBV*) for scenario $Re–LD_{A}–SNP.$ All values are expressed in units of $\sigma_{A}\left(anc\right).$$\sigma_{A}^{2}\left(anc\right)$, mean additive genetic variance; *LD_A_*, ancestral linkage disequilibrium; *LR*, long-range linkage disequilibrium; *Re*, related; SNP, single nucleotide polymorphism; *SR*, short-range linkage disequilibrium.

In general, increasing the number of recombinations $N_{R}$ resulted in a decrease of $r_{g,\hat{g}}$ (C=0, Figure 6), except for scenario $Un–LD_{A}–SNP,$ where $r_{g,\hat{g}}$ stayed nearly constant. Increasing $N_{R}$ in scenario $Re–LD_{A}–Ped$ resulted in the strongest decline in $r_{g,\hat{g}}$ of all scenarios, except if $N_{p}=2,$ where it remained constant. For scenario $Re–LD_{A}–SNP,$ increasing $N_{R}$ from 1 to 5 slightly increased long-term ∑ΔG in C=30 for selection based on *TBV*, but not notably for selection based on *EBV* (Figure 7). The $\sigma_{A}^{2}$ in C=0 was not affected by $N_{R}$ (Figure S4A).

## Discussion

In plant breeding, small effective population sizes that result from a small number of population parents crucially influence the information sources contributing to $r_{g,\hat{g}}$ in a single cycle of GS. For a large number of parents, $LD_{A}$ and pedigree relationships are the driving forces of accuracy, whereas for few parents, cosegregation between QTL and SNPs dominates. While exploitation of information from cosegregation leads to high accuracy, it is unclear how this affects persistency of $r_{g,\hat{g}}$ across selection cycles. Moreover, genetic gain depends on the available genetic variance, which is expected to be reduced for a small number of parents, as opposed to the trend expected for $r_{g,\hat{g}}$. Although persistency and genetic gain in GS have been previously studied, the important situation of the very small effective population sizes in plant breeding, where cosegregation plays a central role, has not been addressed. Hence, the purpose of the present study was to investigate the contributions of the information sources to persistency of $r_{g,\hat{g}}$ and genetic gain across multiple cycles of recurrent GS in synthetic populations, depending on the number of parents.

### Persistency of prediction accuracy across cycles

The persistency of $r_{g,\hat{g}}$ in GS is of crucial importance for practical breeding, because it determines the number of generations that can be employed until retraining of the prediction equation becomes necessary. Thus, it affects the optimum design of a breeding program using recurrent GS and its costs and efficiency compared to phenotypic RS. In agreement with previous studies, we observed a substantial drop in $r_{g,\hat{g}}$ in scenario $Re–LD_{A}–SNP,$ especially after the first cycle (Figure 4). It was hypothesized that this decline is due to a loss of information from pedigree relationships captured by SNPs (Habier *et al.* 2007; Wolc *et al.* 2011b, 2016). In support of this explanation, we observed $r_{g,\hat{g}}$ to plummet after the first cycle in scenario $Re–LD_{A}–Ped$ and this can be attributed to two reasons. First, even without directional selection, the variation in pedigree relationships between the *TS* and *RSC* erodes as the number of generations between both increases (Figure S5C, selection based on *RBV*). Second, selection based on pedigree relationships favors the choice of candidates closely related to one another (Quinton *et al.* 1992; Daetwyler *et al.* 2007), as verified by the substantial increase in inbreeding and the reduced variation in pedigree relationships (Figure S5, A and C), making the breeding population already genetically narrow after only one selection cycle. This causes EBVs to be more similar to each other and hence, also $r_{g,\hat{g}}$ is severely reduced, although the top pedigree relationships between the *TS* and *RSC* individuals increase (Figure S5B). Conversely, selection on *TBV* (corresponding to phenotypic selection with $h^{2}=1$) imposes less inbreeding (Figure S5A), because candidates can have equally high breeding values without necessarily being closely related, which results in the selection of clusters of closely related candidates (Figure S8).

The strong drop of $r_{g,\hat{g}}$ in scenario $Re–LD_{A}–Ped$ for selection based on *EBV* might suggest that pedigree relationships only contribute for one or at least very few generations to $r_{g,\hat{g}}$ of scenario $Re–LD_{A}–SNP.$ However, it has to be taken into account that cosegregation of SNPs and QTL allows capturing of Mendelian sampling (Daetwyler *et al.* 2007), which reduces the selection pressure on pedigree relationships and in turn increases persistency of $r_{g,\hat{g}}$ in scenario $Re–LD_{A}–SNP.$ The effect of reduced selection pressure on pedigree relationships can be inferred from scenario $Re–LD_{A}–Ped$ under selection based on *RBV*, where essentially all selection pressure was removed and individuals were selected irrespective of their ancestry. Here, $r_{g,\hat{g}}$ showed a much slower decay compared to selection based on *EBV* (Figure 4). This suggests that in scenario $Re–LD_{A}–SNP$ with selection based on *EBV*, pedigree relationships probably contribute longer to $r_{g,\hat{g}}$ than indicated by $Re–LD_{A}–Ped$ (selection based on *EBV*).

It was previously shown that information from $LD_{A}$ is highly persistent across generations (Habier *et al.* 2007). In synthetics, the observed LD largely corresponds to $LD_{A}$ only if $N_{p}$ is large, which implies that $LD_{A}$ mainly contributes to $r_{g,\hat{g}}$ for large $N_{p}$ (Schopp *et al.* 2017). Consistent with these findings, for large $N_{p}$ (*e.g.*, 16) $LD_{A}$ was the dominant information source across selection cycles, as verified by the strong reduction in $r_{g,\hat{g}}$ when $LD_{A}$ was artificially removed from scenario $Re–LD_{A}–SNP$ as in $Re–LE_{A}–SNP$ (Figure 4). Conversely, for small $N_{p}$, the representation of $LD_{A}$ in the synthetics is hampered by randomly created sample LD when selecting the parents, which raises the question how this influences persistency of $r_{g,\hat{g}}$ for small $N_{p}.$ Our results show that for $N_{p}=4,$ the persistency of $r_{g,\hat{g}}$ in scenario $Re–LD_{A}–SNP$ was even higher than compared with $N_{p}=16$ where it decreased more strongly, even though the contribution of $LD_{A}$ was markedly reduced (the drop of $r_{g,\hat{g}}$ in scenario $Re–LE_{A}–SNP$ was larger for $N_{p}=4$ than $N_{p}=16$) compared to $N_{p}=16.$ This implies that sample LD and therefore information from cosegregation behaves similarly to $LD_{A}$ regarding the decay of information across selection cycles. The strong conservation of $LD_{A}$ can be directly assessed from scenario $Un–LD_{A}–SNP,$ where *TS* and *RSC* are unrelated and $LD_{A}$ was the only information source (Figure 4). Here, the decay of $r_{g,\hat{g}}$ was generally small, and if selection was based on *RBV* it was even diminutive, indicating that recombination between QTL and SNPs only marginally drives ancestral LD structures of the *TS* and the *RSC* apart. Even if cosegregation information dominates over $LD_{A}$ in the case of small $N_{p}$ (*e.g.*, 4), $LD_{A}$ still substantially contributes to $r_{g,\hat{g}},$ especially in later selection cycles (Figure 4, $Re–LD_{A}–SNP$ *vs.* $Re–LE_{A}–SNP$).

The genomic prediction methodology used can also have a bearing on the exploitation of the sources of information, which was not considered in this study. Previous research indicated that (Bayesian) variable selection methods are better suited to capture information from $LD_{A}$ compared to GBLUP, especially if traits are oligogenic and individual QTL have strong effects (Habier *et al.* 2007, 2013; Zhong *et al.* 2009). Therefore, we expect that such methods are advantageous in situations where $r_{g,\hat{g}}$ heavily relies on information from $LD_{A},$ as is the case for large $N_{p}$ or if *TS* and *RSC* are unrelated.

### Steady state cumulative genetic gain

In any population advanced by RS, the cumulative increase in overall performance is of central interest to breeders. Here, we continued RS until cycle C=30, where further increases in ∑ΔG were only marginal because either $\sigma_{A}^{2}$ was depleted (Figure S6) and/or $r_{g,\hat{g}}$ was near zero (Figure 4). This approach allowed for direct comparisons between ∑ΔG for different scenarios and conclusions were not contingent on the amount of $\sigma_{A}^{2}$ left.

Increasing $N_{p}$ leads to an asymptotic increase in the initially available $\sigma_{A}^{2},$ which was independent of the ancestral population in our simulation (Figure S7). According to the breeder’s equation, increasing $\sigma_{A}^{2}$ results in higher genetic gain, which partially explains the increase in ∑ΔG for larger $N_{p}.$ However, besides higher $\sigma_{A}^{2},$ differential contributions of the three sources of information to $r_{g,\hat{g}}$ play a major role. In scenario $Re–LD_{A}–Ped,$ ∑ΔG was relatively constant from medium $N_{p}\geq 8$ on (Figure 3), which is presumably the result of the counterbalancing effects of a slight increase in $\sigma_{A}^{2}$ and a moderate decrease in $r_{g,\hat{g}}$ with increasing $N_{p}.$ As pointed out by Schopp *et al.* (2017), increasing $N_{p}$ from medium to large values decreases the frequency of close relatives between *TS* and *RSC* and hence, reduces $r_{g,\hat{g}}$ (Figure S3). The contribution of pedigree relationships to long-term genetic gain in scenario $Re–LD_{A}–SNP$ should therefore be relatively constant for medium to large $N_{p}.$ As the contribution of cosegregation to $r_{g,\hat{g}}$ decreases with larger $N_{p},$ ∑ΔG of scenario $Re–LE_{A}–SNP$ strongly declined. Conversely, ∑ΔG of scenario $Un–LD_{A}–SNP$ strongly increased with larger $N_{p}$ due to more information from $LD_{A}.$ Given that there is sufficient $LD_{A}$ present in the ancestral population (*LR*), both effects largely compensate for each other and hence, ∑ΔG in scenario $Re–LD_{A}–SNP$ appears to be insensitive to changes in $N_{p}$ beyond four parents for *LR* (Figure 3). When there is not sufficient $LD_{A}$ as applies to *SR*, increasing information due to $LD_{A}$ can no longer compensate for the loss in cosegregation information and therefore ∑ΔG in $Re–LD_{A}–SNP$ decreased for higher $N_{p}.$ Although we considered ∑ΔG close to its steady state, it is important to note that the essential trends in ∑ΔG are already apparent for as few as two selection cycles (Figure S2), which implies that our observations do not only apply to the situation of extreme long-term selection without retraining, but also to few selection cycles.

### Influence of TS size and SNP density

We found that increasing $N_{TS}$ leads to higher persistency of $r_{g,\hat{g}}$ in early selection cycles for scenarios with pedigree relationship between *TS* and *RSC* ($P^{TS}=P^{RSC},$ Figure 5). This is because, for a given $N_{p},$ increasing $N_{TS}$ enhances the probability of obtaining *TS* individuals that share an exceptionally large portion of their genome with the *RSC* individuals due to Mendelian sampling and because of similarities between individuals due to $LD_{A}.$ Hence, for small $N_{TS}$ there is a higher reliance on information from pedigree relationships (Jannink *et al.* 2010; Schopp *et al.* 2017) that quickly erodes under directional selection. For large $N_{TS},$ there is a higher weight on information from cosegregation and $LD_{A},$ which in turn increases the persistency of $r_{g,\hat{g}}.$ This shift in emphasis also entails reduced inbreeding, especially in early selection cycles (results not shown), in agreement with the findings of Jannink (2010). Therefore, if a prediction equation is to be used for multiple cycles, $N_{TS}$ should be chosen large enough to not only guarantee high initial $r_{g,\hat{g}},$ but also high persistency of $r_{g,\hat{g}}$ and reduced inbreeding in order to improve genetic gain from GS. Increasing SNP density from 0.125 to 2.5 ${\text{cM}}^{-1},$ corresponding to ∼250 and 5000 SNPs in the case of maize, led to an increase in the persistency of $r_{g,\hat{g}}$ (Figure 5), which is in concordance with previous studies (Solberg *et al.* 2009; Sonesson and Meuwissen 2009). Higher SNP density theoretically affects all three sources of information, but its influence should be strongest on $LD_{A}$ and cosegregation because they rely on physical proximity of SNPs and QTL. If the SNP density is extremely low (*e.g.*, $0.125\;\;{\text{cM}}^{-1}$), it is unlikely that SNPs and QTL are tightly linked and hence, SNPs mainly capture pedigree relationships, whereas $LD_{A}$ and cosegregation play only subordinate roles. Therefore, high SNP density improves persistency of $r_{g,\hat{g}}$over generations, because information from both $LD_{A}$ (Figure 5, $N_{p}=16$) and cosegregation (Figure 5, $N_{p}=2$) are less prone to decay, compared to pedigree relationships. The highest SNP density we investigated was 2.5 ${\text{cM}}^{-1},$ which is relatively low compared to what is nowadays available in many plant species. However, because of the strong influence of cosegregation in synthetics that are produced from a low to intermediate number of parents, we would expect that little can be gained by further increasing SNP density, especially if long-range LD_A_ is present, as can be assumed for elite germplasm in practical applications. However, the situation can be quite different for large $N_{p}$ and if there is only short-range LD_A_ in the ancestral population, which rapidly increases the need for higher SNP densities.

### Influence of the number of recombination generations

We hypothesized that larger $N_{R}$ might lead to enhanced long-term ∑ΔG by virtue of a stronger fragmentation of chromosomes in the synthetic. Actually, the average length of chromosomal segments of unique parental origin decreased from ∼66 cM for $N_{R}=1$ to 30 cM ($N_{p}=2$) and 20 cM ($N_{p}=16$) for $N_{R}=5$ (Figure S4B). However, as information from pedigree relationships strongly declined with increasing $N_{R}$ (Figure 6, scenario $Re–LD_{A}–Ped$), $r_{g,\hat{g}}$ in C=0 generally decreased in scenario $Re–LD_{A}–SNP.$ Conversely, the decline of information contributed by $LD_{A}$ with increasing $N_{R}$ was negligible (scenario$\;Un–LD_{A}–SNP$). Decreasing selection accuracy reduces ∑ΔG, which can conceal the positive effect of higher genome fragmentation. Analysis of the latter factor alone is possible with selection regime *TBV*, where selection accuracy was always constant and equal to one, regardless of $N_{R}.$ Here, we found higher ∑ΔG for $N_{R}=5$ compared to $N_{R}=1$ (Figure 7) because finer fragmentation promotes occurrence of genotypes with favorable allele combinations for selection. This is accompanied by a reduced coselection of QTL, such that more QTL stay polymorphic and therefore $\sigma_{A}^{2}$ remains higher in advanced selection cycles. The positive effect of $N_{R}$ on ∑ΔG under selection on *TBV* increased with increasing $N_{p},$ presumably because larger $N_{p}$ results in even finer genome fragmentation (Figure S4B). For selection regime *EBV*, ∑ΔG in C=30 was not higher for $N_{R}=5$ than for $N_{R}=1,$ suggesting that positive and negative effects of recombination cancelled out each other. For ancestral population *SR*, ∑ΔG was even slightly lower for $N_{R}=5,$ because compared to *LR*, stochastic dependency between QTL is relatively low from the beginning and hence, higher fragmentation has only a minor effect. A special situation existed for $N_{p}=2,$ which is explained in File S1.

It is noteworthy that in our simulations the initial $\sigma_{A}^{2}$ (C=0) was unaffected by $N_{R},$ although strong sample LD between QTL was broken up. In reality, ancestral populations (corresponding to source germplasms in breeding) generally underwent some sort of directional selection, which can theoretically cause a reduction in $\sigma_{A}^{2}$ due to the Bulmer effect (Bulmer 1971; Long *et al.* 2011). This hidden part of $\sigma_{A}^{2}$ attributable to negative LD between causal loci can be recovered by recombination, which might lead to an increase in ∑ΔG for $N_{R}>1.$

### Implications for practical applications

At the start of any breeding program employing GS with the goal of improving quantitative traits, breeders have to make a number of crucial decisions, including the source germplasm, parents, and mating scheme used to develop the breeding population. Further decisions specific to GS concern the $N_{TS}$ and marker density. All of these factors influence the importance of the three information sources in GS and thereby have ramifications on the success of the breeding program.

The choice of the source germplasm crucially determines the improvement potential for the target trait (Fountain and Hallauer 1996), because it determines the genetic diversity and linkage disequilibrium (*i.e.*, $LD_{A}$), which are both of central importance for the success of GS. Our study demonstrates that information from $LD_{A}$ generally offers high persistency across selection cycles in synthetics, irrespective of $N_{p}.$ Hence, $LD_{A}$ is particularly important for ensuring sustained genetic progress during the breeding program. However, the contribution of $LD_{A}$ to genetic gain is itself highly dependent on $N_{p}.$ Whereas for large $N_{p},$ LD in synthetics adequately represents $LD_{A},$ small $N_{p}$ generates sample LD and, in turn, cosegregation that dominates LD in synthetics. Cosegregation has a similarly high persistency as $LD_{A},$ but it can only contribute to genetic gain if *TS* and selection candidates are related by having parents in common. However, it must be taken into account that reducing $N_{p}$ also reduces the initially available genetic variance for breeding, thereby impairing ∑ΔG. In essence, high persistency of $r_{g,\hat{g}}$ and thereby prolonged genetic progress may be achieved irrespective of $N_{p},$ but if $N_{p}$ is large, substantial $LD_{A}$ is required.

Pedigree relationships also contribute to predictive information for $N_{p}>2,$ and harnessing pedigree information has been recommended to achieve high $r_{g,\hat{g}}$ in GS (*e.g.*, Wolc *et al.* 2011a). Frequent retraining of the prediction equation, at best in every generation, would be required to optimally exploit pedigree relationships because information from them rapidly erodes over generations, especially under directional selection. In addition, selection using pedigree relationships increases the rate of inbreeding due to intraclass correlation of EBV for members of the same family and their coselection (Daetwyler *et al.* 2007), a result that is well known in animal breeding (Belonsky and Kennedy 1988) and was confirmed in our study for synthetics in plant breeding (Figure S5A). A high rate of inbreeding is undesirable in long-term selection, because genetic diversity is rapidly depleted and eventually ∑ΔG is compromised. In GS, it was shown that molecular markers not only capture deviations of genomic relationships from pedigree relationships, but also the pedigree relationships themselves (Habier *et al.* 2007), *i.e.*, the latent family structure in the case of synthetics. Therefore, the same concerns as for pedigree-based selection partially apply to GS, so that GS is also prone to selection of close relatives and inbreeding (Jannink 2010). If the breeding objective is long-term ∑ΔG, as classically targeted by RS in genetically broad-based populations (Hallauer and Carena 2012), corresponding to large $N_{p}$ in our study, deliberate avoidance of using pedigree relationships might be desirable for maximizing long-term ∑ΔG.

There are different possibilities to reduce the influence of pedigree relationships. Increasing both $N_{TS}$ and marker density leads to an improved capturing of Mendelian sampling and similarities between individuals due to $LD_{A},$ which reduces the reliance on pedigree relationships and in turn reduces inbreeding. Another possibility could be modeling information from $LD_{A},$ cosegregation (Calus *et al.* 2008; Legarra and Fernando 2009), and pedigree relationships in a joint linear mixed model in an attempt to isolate information from pedigree relationships. Alternatively, one could modify the mating scheme used for generating the synthetic. Additional generations of recombination successfully decreased strong variation in pedigree relationships between individuals, but only up to $N_{p}≅5$ where a baseline level was reached (Figure S4C). Mating schemes as employed for establishing the Multi-parent Advanced Generation Intercrosses (MAGIC) largely avoid population substructure and pedigree relationships, while they complement the favorable properties of synthetics such as high genetic diversity and elevated minor allele frequencies with a fine-grained mosaic of the genome (compare Dell’Acqua *et al.* 2015; Holland 2015). Thus, they potentially represent ideal candidates for long-term recurrent GS, but this warrants further research.

## Supplementary Material

Supplemental material is available online at www.g3journal.org/lookup/suppl/doi:10.1534/g3.116.036582/-/DC1.
