# Supplementary material for: Persistency of Prediction Accuracy and Genetic Gain in Synthetic Populations Under Recurrent Genomic Selection
Source: G3 (Bethesda). 2017 Jan 4;7(3):801–11. doi: 10.1534/g3.116.036582 (PMC5345710; doi:10.1534/g3.116.036582)
Supplement: Supplementary file 1 [file 801FigureS1.pdf]

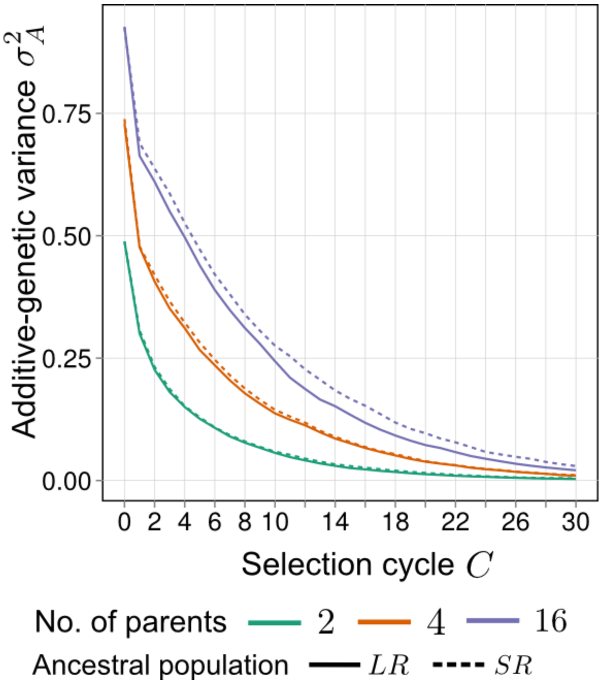

Figure S1 Additive genetic variance σ_A^2 in scenario Re– LD_A– SNP under recurrent genomic selection across $C = 0, 1, \dots, 30$ selection cycles for synthetics produced from $N_p = 2, 4, 16$ parents taken from ancestral populations *SR* or *LR*. Values in are expressed in units of $\sigma_A^2(anc)$, respectively.
