# Supplementary material for: Persistency of Prediction Accuracy and Genetic Gain in Synthetic Populations Under Recurrent Genomic Selection
Source: G3 (Bethesda). 2017 Jan 4;7(3):801–11. doi: 10.1534/g3.116.036582 (PMC5345710; doi:10.1534/g3.116.036582)
Supplement: Supplementary file 2 [file 801FigureS2.pdf]

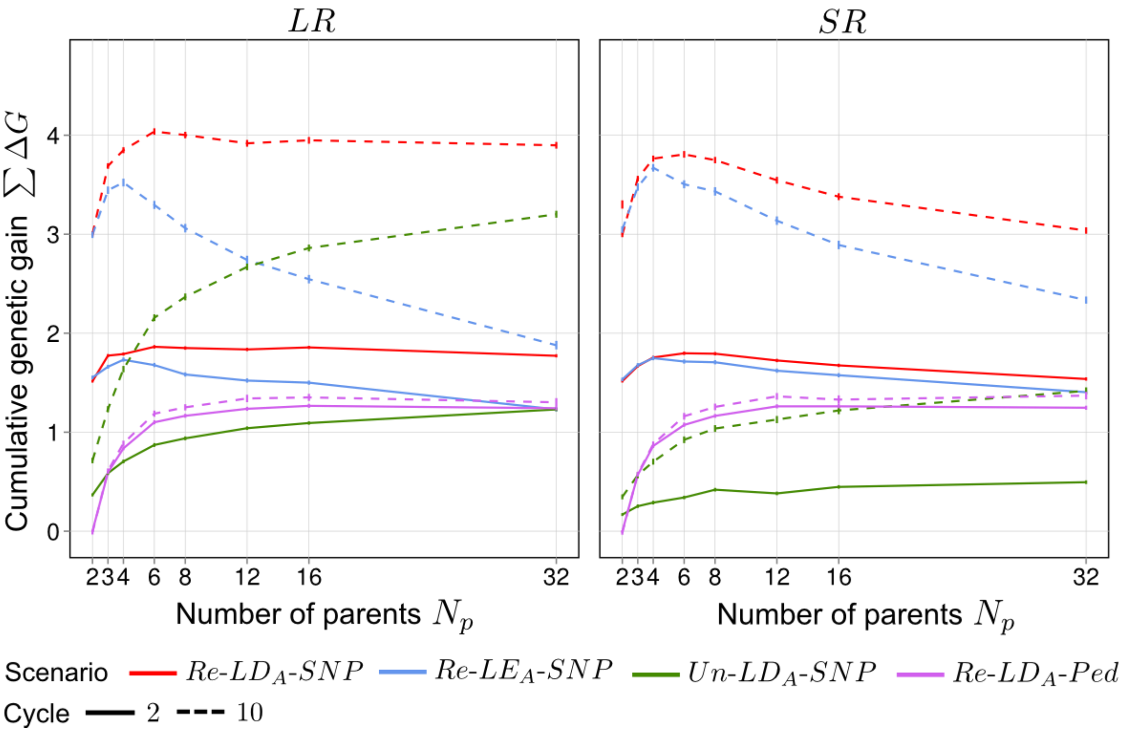

Figure S2 Average cumulative genetic gain $\sum \Delta G$ under recurrent genomic selection in different selection cycles for synthetics produced from different numbers of parents N_p taken from ancestral populations *SR* or *LR*. All values are expressed in units of $\sigma_A(anc)$.
