# Supplementary material for: Persistency of Prediction Accuracy and Genetic Gain in Synthetic Populations Under Recurrent Genomic Selection
Source: G3 (Bethesda). 2017 Jan 4;7(3):801–11. doi: 10.1534/g3.116.036582 (PMC5345710; doi:10.1534/g3.116.036582)
Supplement: Supplementary file 3 [file 801FigureS3.pdf]

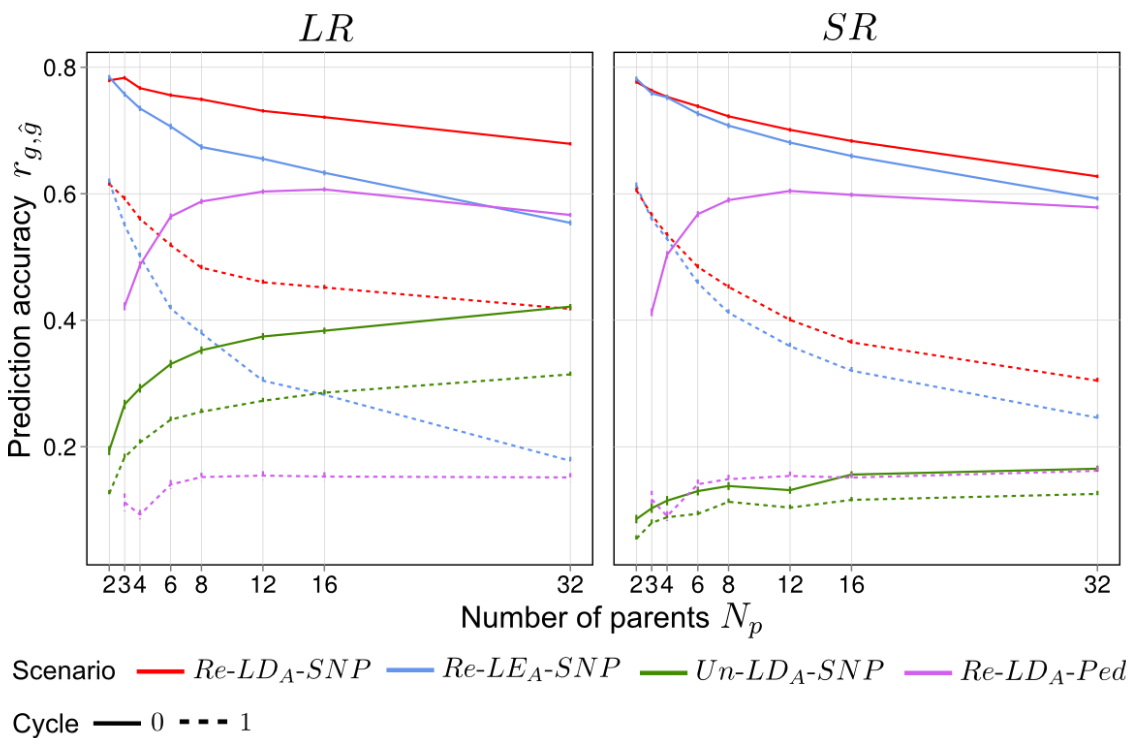

Figure S3 Average prediction accuracy $r_{g,\hat{g}}$ in selection cycles $C = 0$ and $C = 1$ for synthetics produced from different numbers of parents N_p taken from ancestral populations *SR* and *LR*.
