# Supplementary material for: Persistency of Prediction Accuracy and Genetic Gain in Synthetic Populations Under Recurrent Genomic Selection
Source: G3 (Bethesda). 2017 Jan 4;7(3):801–11. doi: 10.1534/g3.116.036582 (PMC5345710; doi:10.1534/g3.116.036582)
Supplement: Supplementary file 4 [file 801FigureS4.pdf]

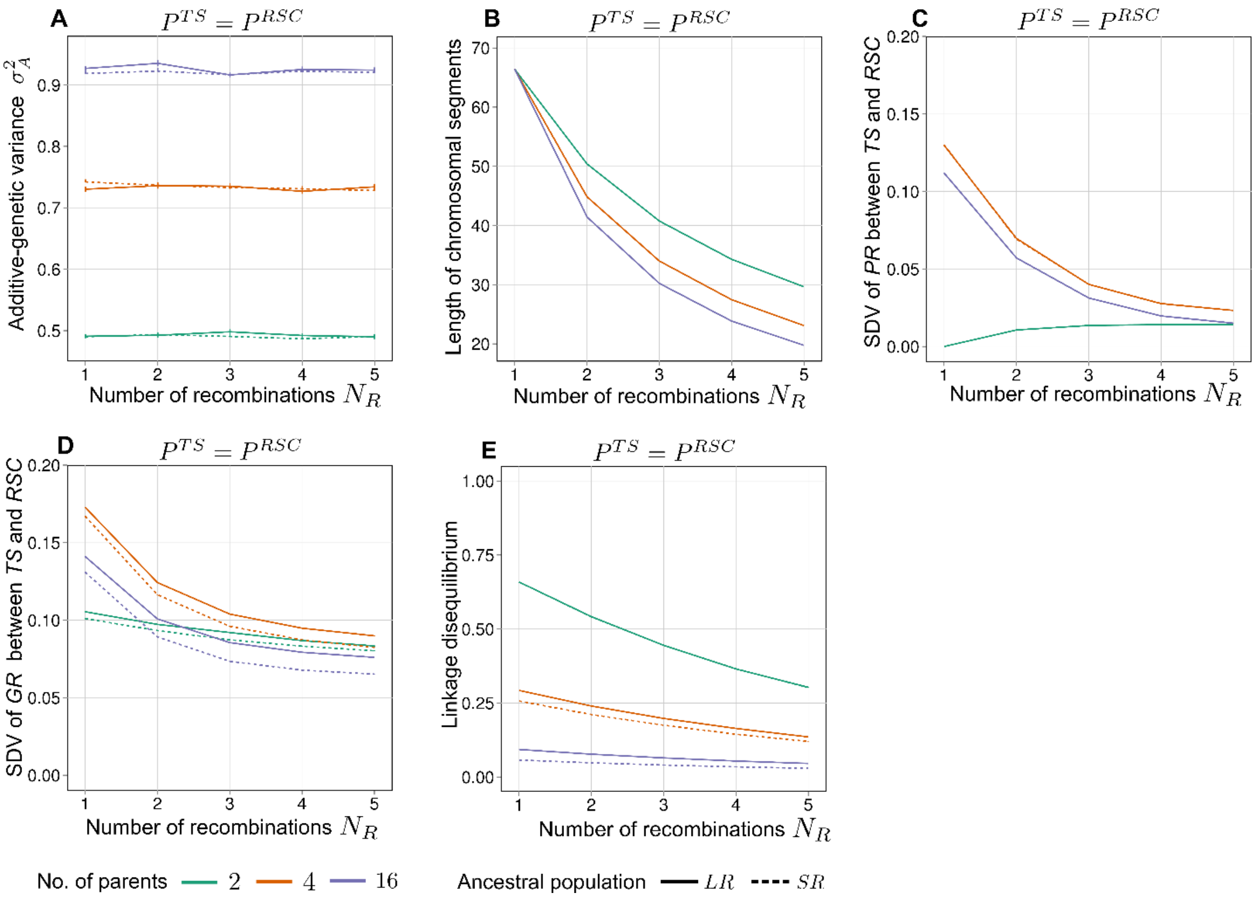

Figure S4 (A) Avareage additive genetic variance σ_A^2, (B) average length of chromosomal segments of parental origin, (C) average standard deviation (SDV) of pedigree relationships (*PR*) between *TS* and *RSC*, (D) average standard deviation of genomic relationships (*GR*) between *TS* and *RSC,* (E) average linkage disequilibrium in selection cycles $C = 0$ for synthetics produced from $N_p = 2, 4, 16$ parents taken from ancestral populations *SR* or *LR*. Values in (A) are expressed in units of $\sigma_A^2(anc)$. Values in (E) refer to QTL-SNP pairs with a genetic distance between 10 and 11 cM. All results refer to averages of scenarios with $P^{TS} = P^{RSC}$, because expected values were identical.
