# Supplementary material for: Persistency of Prediction Accuracy and Genetic Gain in Synthetic Populations Under Recurrent Genomic Selection
Source: G3 (Bethesda). 2017 Jan 4;7(3):801–11. doi: 10.1534/g3.116.036582 (PMC5345710; doi:10.1534/g3.116.036582)
Supplement: Supplementary file 5 [file 801FigureS5.pdf]

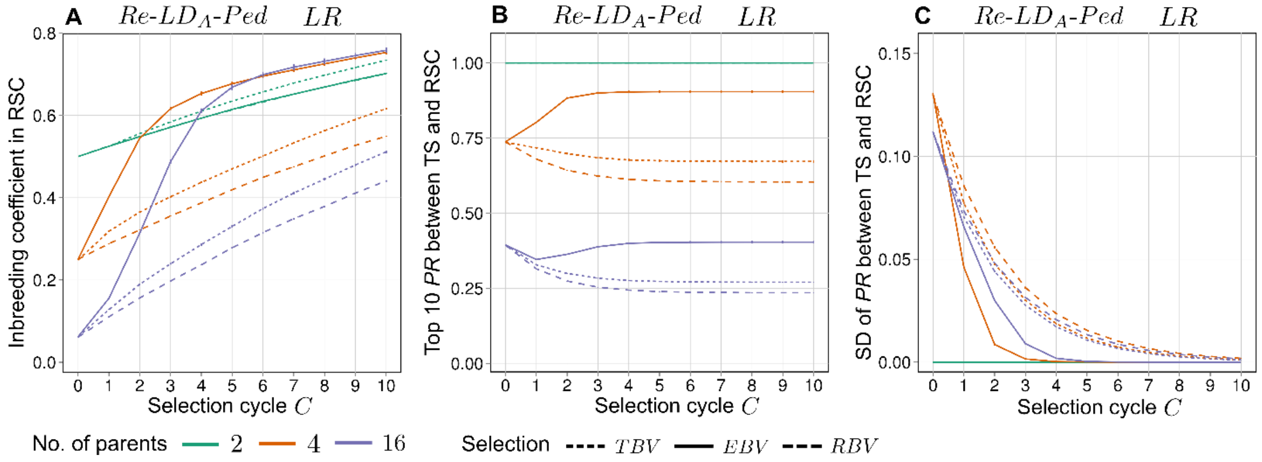

Figure S5 (A) Average inbreeding coefficient in the *RSC*, (B) average of top 10 pedigree relationships (*PR*) between each *RSC* individual and the TS, (C) average standard deviation of pedigree relationships in *RSC* under recurrent genomic selection across selection cycles $C = 0, 1, \dots, 10$ for synthetics produced from $N_p = 2, 4, 16$ parents taken from ancestral population *LR*. Results refer to scenario $Re\text{-}LD_A\text{-}Ped$.
