# Supplementary material for: Persistency of Prediction Accuracy and Genetic Gain in Synthetic Populations Under Recurrent Genomic Selection
Source: G3 (Bethesda). 2017 Jan 4;7(3):801–11. doi: 10.1534/g3.116.036582 (PMC5345710; doi:10.1534/g3.116.036582)
Supplement: Supplementary file 6 [file 801FigureS6.pdf]

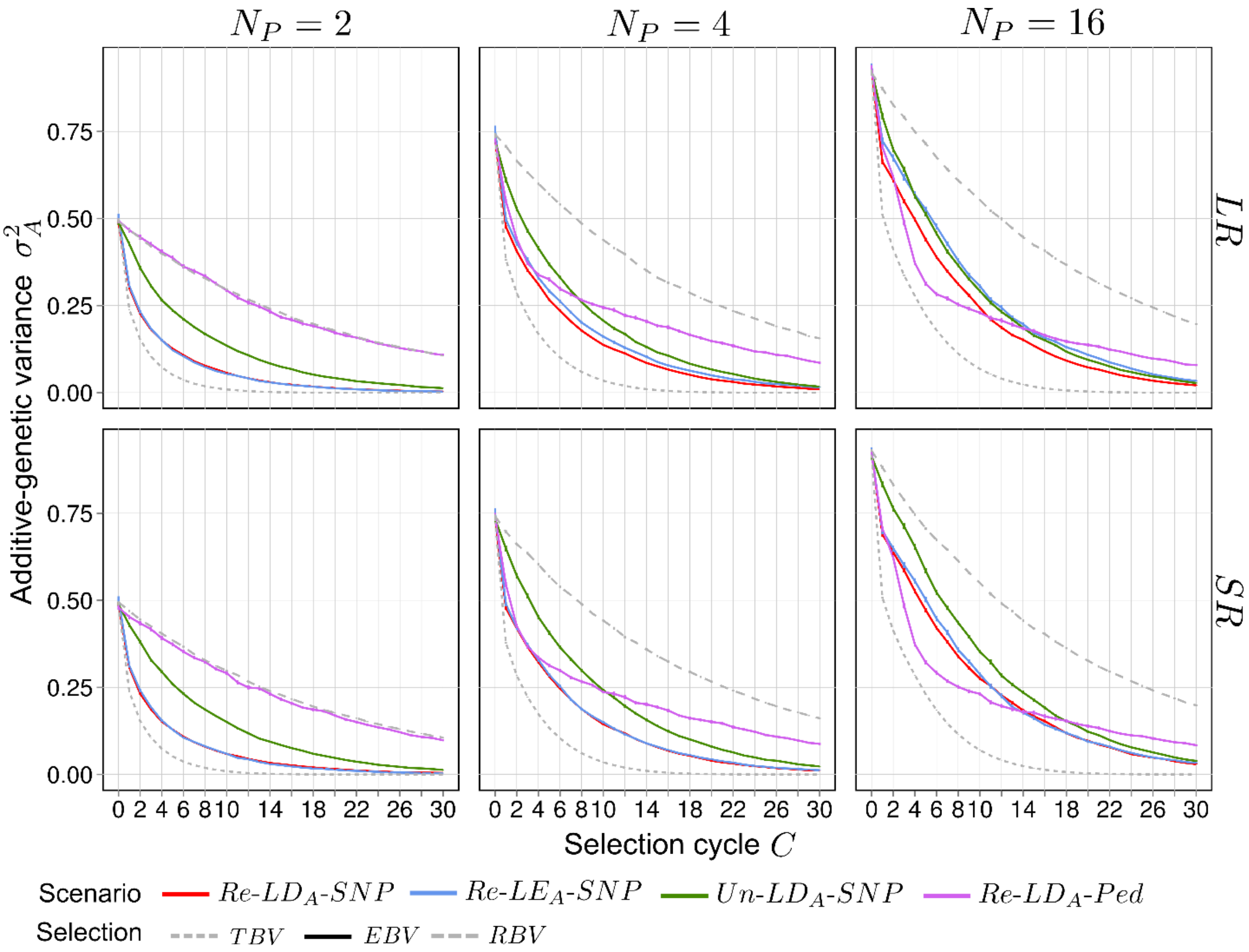

Figure S6 Average additive genetic variance σ_A^2 under recurrent genomic selection across selection cycle $C = 0, 1, \ldots, 30$ for synthetics produced from $N_p = 2, 4, 16$ parents taken from ancestral populations *SR* and *LR*. Selection of candidates was based on either true breeding values (*TBV*), random breeding values (*RBV*) or estimated breeding values (*EBV*) under different information scenarios. Values in are expressed in units of $\sigma_A^2(anc)$. Because σ_A^2 has identical expected values across scenarios for selection based on *TBV* and *RBV*, the means across scenarios are shown.
