# Supplementary material for: Persistency of Prediction Accuracy and Genetic Gain in Synthetic Populations Under Recurrent Genomic Selection
Source: G3 (Bethesda). 2017 Jan 4;7(3):801–11. doi: 10.1534/g3.116.036582 (PMC5345710; doi:10.1534/g3.116.036582)
Supplement: Supplementary file 7 [file 801FigureS7.pdf]

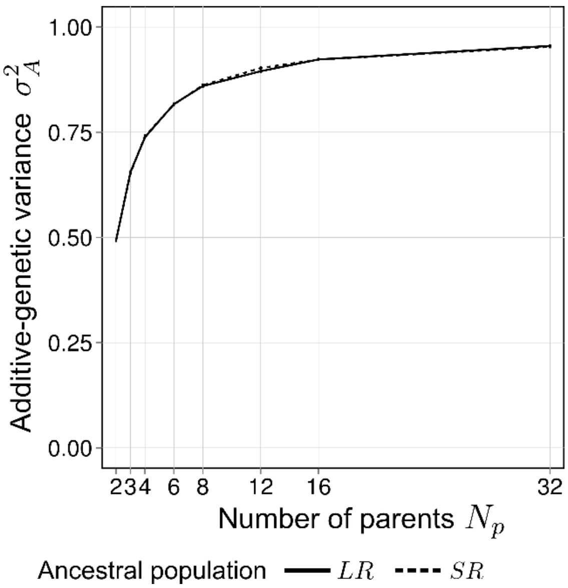

Figure S7 Additive genetic variance σ_A^2 in selection cycle $C = 0$ for synthetics produced from different numbers of parents N_p taken from ancestral populations *SR* and *LR*. Values in are expressed in units of $\sigma_A^2(anc)$.
