# Supplementary material for: Persistency of Prediction Accuracy and Genetic Gain in Synthetic Populations Under Recurrent Genomic Selection
Source: G3 (Bethesda). 2017 Jan 4;7(3):801–11. doi: 10.1534/g3.116.036582 (PMC5345710; doi:10.1534/g3.116.036582)
Supplement: Supplementary file 8 [file 801FigureS8.pdf]

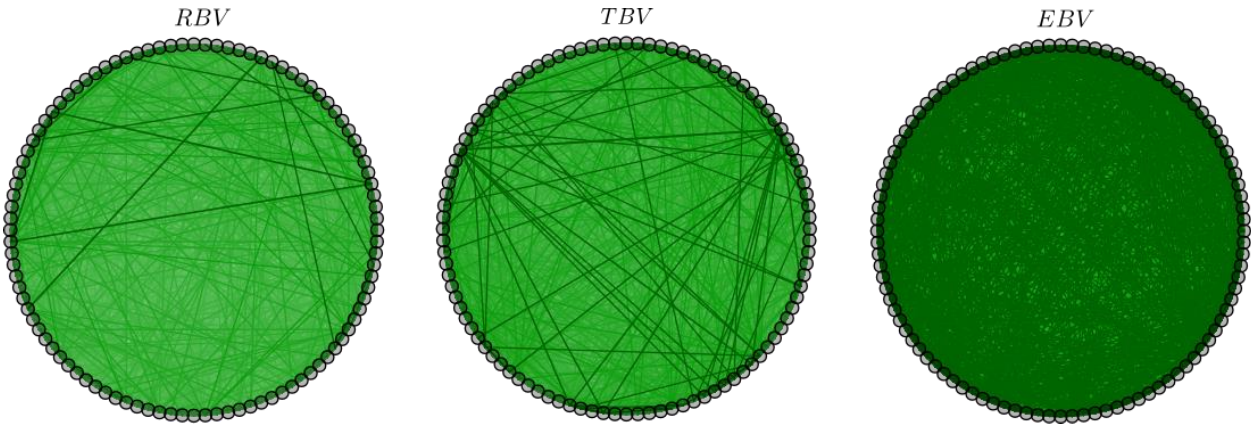

Figure S8 Visualization of additive relationships (*AR*) among 100 selection candidates after a single round of selection either based on random breeding values (*RBV*), estimated breeding values (*EBV)* or true breeding values (*TBV*) for scenario $Re\text{–}\,LD_A\text{–}\,Ped$ and for number of parents $N_p = 4$. The strength of the *AR* is represented by the thickness of the lines connecting selection candidates. The figure was computed using the *R*-package *qgraph* based on a single representative simulation run.
