# Supplementary material for: Persistency of Prediction Accuracy and Genetic Gain in Synthetic Populations Under Recurrent Genomic Selection
Source: G3 (Bethesda). 2017 Jan 4;7(3):801–11. doi: 10.1534/g3.116.036582 (PMC5345710; doi:10.1534/g3.116.036582)
Supplement: Supplementary file 9 [file 801FileS1.pdf]

File S1: Special case of synthetics with $N_p = 2$.

Synthetics with $N_p = 2$ and $N_R = 1$ represent a special case, because in scenario $Re{-}LD_A{-}Ped$, $r_{g,\hat{g}}$ could not be calculated as all *TS* and *RSC* individuals are full-sibs and, therefore, all predicted breeding values are identical. For $N_R \geq 2$, variation in pedigree relationships between the *TS* and the *RSC* emerges, resulting in $r_{g,\hat{g}} \geq 0.2$ (Figure 6). Surprisingly, $r_{g,\hat{g}}$ in scenario $Re{-}LD_A{-}SNP$ was largely unaffected by recombination, most likely because the newly generated variation in pedigree relationships was small compared with the variation in Mendelian sampling already present for $N_R = 1$. The much smaller standard deviation of pedigree relationships than genomic relationships between the *TS* and *RSC* shown in Figures S4C and S4D supports this explanation.
