# Supplementary material for: Persistency of Prediction Accuracy and Genetic Gain in Synthetic Populations Under Recurrent Genomic Selection
Source: G3 (Bethesda). 2017 Jan 4;7(3):801–11. doi: 10.1534/g3.116.036582 (PMC5345710; doi:10.1534/g3.116.036582)
Supplement: Supplementary file 11 [file 801FileS2.docx]

File S2: R code used to conduct the simulations. (.zip, 23 KB)

Available for download as a .zip file at:

http://www.g3journal.org/lookup/suppl/doi:10.1534/g3.116.036582/-/DC1/FileS2.zip
