# Supplementary material for: Persistency of Prediction Accuracy and Genetic Gain in Synthetic Populations Under Recurrent Genomic Selection
Source: G3 (Bethesda). 2017 Jan 4;7(3):801–11. doi: 10.1534/g3.116.036582 (PMC5345710; doi:10.1534/g3.116.036582)
Supplement: Supplementary file 12 [file 801TableS1.pdf]

**Table S1** Characterization of the four scenarios analyzed in this study to assess the contributions of (i) pedigree relationships among individuals in the training set (*TS*) and recurrent selection candidates (*RSC*) captured by SNPs, (ii) co-segregation of QTL and SNPs, and (iii) ancestral linkage disequilibrium ($LD_A$) between QTL and SNPs to persistency of prediction accuracy $r_{g,\hat{g}}$ and cumulative genetic gain $\sum \Delta G$ in synthetics. Note that pairs of SNP markers and pairs of QTL were always allowed to be in $LD_A$.

| Scenario | TS and RSC are | QTL and SNPs in | Relationship matrix calculated from | Type |
|---|---|---|---|---|
| $Re\text{–}LD_A\text{–}SNP$ | Related ($P_{TS} = P_{RSC}$) | $LD_A$ | SNPs | Realistic |
| $Re\text{–}LD_A\text{–}Ped$ | | $LD_A$ | Pedigree records | Realistic |
| $Re\text{–}LE_A\text{–}SNP$ | | $LE_A$ | SNPs | Artificial |
| | | | | |
| $Un\text{–}LD_A\text{–}SNP$ | Unrelated ($P_{TS} \cap P_{RSC} = \emptyset$) | $LD_A$ | SNPs | Realistic |
